# Predictive value of the composition of the vaginal microbiota in bacterial vaginosis, a dynamic study to identify recurrence-related flora

**DOI:** 10.1038/srep26674

**Published:** 2016-06-02

**Authors:** Bingbing Xiao, Xiaoxi Niu, Na Han, Ben Wang, Pengcheng Du, Risu Na, Chen Chen, Qinping Liao

**Affiliations:** 1 Department of Obstetrics and Gynecology, Peking University First Hospital, Xi’anmen Street, Beijing 100034, China; 2 State Key Laboratory for Infectious Disease Prevention and Control, National Institute for Communicable Disease Control and Prevention, Chinese Center for Disease Control and Prevention, Beijing 102206, China; 3 Department of Obstetrics and Gynecology, Beijing Tsinghua Changgung Hospital, Beijing 102218, China; 4 Beijing Key Laboratory of Emerging Infectious Diseases, Beijing 100015, China; 5 Institute of Infectious Diseases, Beijing Ditan Hospital, Capital Medical University, Beijing 100015, China

## Abstract

Bacterial vaginosis (BV) is a highly prevalent disease in women, and increases the risk of pelvic inflammatory disease. It has been given wide attention because of the high recurrence rate. Traditional diagnostic methods based on microscope providing limited information on the vaginal microbiota increase the difficulty in tracing the development of the disease in bacteria resistance condition. In this study, we used deep-sequencing technology to observe dynamic variation of the vaginal microbiota at three major time points during treatment, at D0 (before treatment), D7 (stop using the antibiotics) and D30 (the 30-day follow-up visit). Sixty-five patients with BV were enrolled (48 were cured and 17 were not cured), and their bacterial composition of the vaginal microbiota was compared. Interestingly, we identified 9 patients might be recurrence. We also introduced a new measurement point of D7, although its microbiota were significantly inhabited by antibiotic and hard to be observed by traditional method. The vaginal microbiota in deep-sequencing-view present a strong correlation to the final outcome. Thus, coupled with detailed individual bioinformatics analysis and deep-sequencing technology, we may illustrate a more accurate map of vaginal microbial to BV patients, which provide a new opportunity to reduce the rate of recurrence of BV.

Bacterial vaginosis (BV) is the most prevalent form of vaginal infection among women of reproductive age, affecting 8–23%, and is the most common etiology of vaginal symptoms prompting women to seek medical care^1^. Several adverse health outcomes are strongly associated with BV, including pelvic inflammatory disease, postpartum endometritis, preterm labor and delivery, and increased susceptibility to infection with *Trichomonas vaginalis*, *Chlamydia trachomatis*, *Vulvovaginal candidiosis*, and HIV^2^,^3^,^4^,^5^,^6^. Although it has been studied for half a century since the term was coined, the etiology and natural history of BV remain elusive^7^. Recent studies of the composition of the vaginal microbiota have provided new clues to the etiology of BV^8^,^9^,^10^,^11^. In patients with BV, the microbiota shift from *Lactobacillus*-dominated in the healthy population to high quantities of commensal anaerobes, such as *Atopobium*, *Gardenerella*, *Prevotella*, and *Papillibacter*, which are considered the major disease-causing agents^12^,^13^. To date, most studies of the vaginal microbiota have focused on distinguishing patients with BV from the general population on the basis of differences at the bacterial species or genus level, identified by various bioinformatics methods and databases^12^. These data have provided information on genera and species that are strongly associated with clinical features of BV, and it is supposed that a complex network plays a comprehensive role in regulating the infection and the immune response^14^. However, few data presented from a continuous follow-up study. In this study, we followed patients at three time points to investigate the composition of their vaginal microbiota, in order to construct a prospective longitudinal summary of the transition of the microbiome during the treatment and recovery period of BV.

Metronidazole and clindamycin are the major antibiotics used in the clinical treatment of BV. These drugs inhibit bacterial growth and significantly change the vaginal microbiome. However, patients with BV present a high rate of recurrence, with cure rates from 44.4–83% after standard treatment^15^,^16^. Moreover, according to general procedures, not all patients are recommended to undergo a follow-up examination unless symptoms reappear, so it is impossible to observe the state of disease progression or changes in the composition of the vaginal microbiota. Among patients with recurrent BV, many may not have recovered completely after treatment, although they have met the criteria of the traditional Amsel’s diagnostic and Nugent score systems after antibiotic use^17^. The proposed quantitative PCR (qPCR) method providing new insight into the composition of bacteria is a useful tool to detect the microbe in clinical diagnosis even in low centration^18^,^19^. Two different teams identified combining to detect the level of *Atopobium vaginae*, *Gardnerella vaginalis*, *Lactobacillus* could early detect BV^18^,^19^. However, limited by the number of pathogens species they detected, we are still hard to illustrate clearer process of dynamic changes of microbiota in BV patients, and then to identify the recurrence.

The development of culture-independent molecular approaches based on high throughput sequencing of 16S rRNA genes (or 16S rDNA) allows us to study the vaginal microbiota sequentially in order to observe the progress of BV^12^. In this study, to examine the vaginal flora at different time points, and to study the relationship between changes in the microbial population before/after antibiotic treatment and the results of therapy, we chose the drug generally recommended for treatment of BV-metronidazole-as the target drug. Using pyrosequencing, we sought to develop an in-depth and accurate understanding of the composition and ecology of the vaginal microbial ecosystem after standard intravaginal treatment. This method was used to identify the potential correlations between the vaginal bacterial flora and the treatment outcome. Furthermore, we proposed the addition of a time point, D7 (6–8 days after the initial visit), as a new examination time. Our findings suggest that using information on the microbiota could provide a much more accurate and dynamic map of the transition of the bacterial flora, which documents the progression of BV, and provides a guide to subsequent clinical treatment.

## Results

### Characterization of women with bacterial vaginosis after active treatment with metronidazole, and at the follow-up visit

To characterize the continuous development of the vaginal microbiome in patients with BV, we performed prospective collection of vaginal swabs from women who were diagnosed with BV and treated with intravaginal metronidazole. We examined the patients at three time points in our study, at D0 (the initial visit), D7 (6–8 days after the initial visit) and D30 (at the 30-day follow-up visit). Among 109 patients with BV who were treated with metronidazole, we performed comprehensive follow-up examinations at all three time points on 73 patients. After excluding 5 patients who were affected with *Vulvovaginal candidiasis* (4 patients) or *Trichomonal vaginitis* (1 patient), 68 women were enrolled in our study. The vaginal microbial community sampled at D0, D7 and D30, in a total of 204 samples, was sequenced to yield 1,622,359 high-quality reads with an average of 8071 (range 3424–18,517) sequences per sample. Three patients were excluded because of a relatively low amount of reads obtained at one or two time points. We found a total of 8967 operation taxonomy units (OTUs) across all the samples, with a range of 54–706 OTUs present in each individual sample. The details of the OTUs are available in Supplemental Table 1. According to the Nugent score at D30, the 65 patients were divided into two groups, 48 patients were in the successfully cured group (SG) and 17 patients in the failed group (FG), and samples from these patients were included in further microbiome analysis.

**Figure 1:**
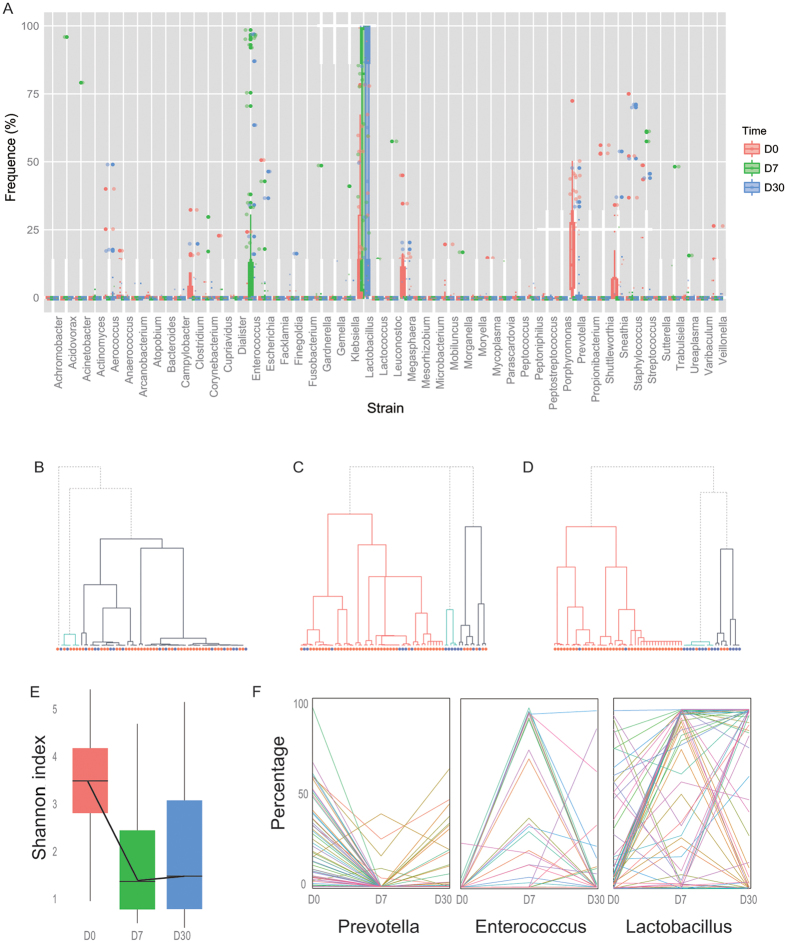
The vaginal bacterial communities in vaginal swabs from patients with bacterial vaginosis (BV). (**A**) The relative abundance of predominant bacterial taxonomic groups at the three time points. Red represents D0 (the initial day), green represents D7 (the 7th day from the initial assessment, 1–3 days after metronidazole treatment) and blue represents D30 (30 days’ follow-up visit). (BCD) Phylogenetic trees based on the bacterial communities at D0, D7 and D30, respectively. The bacterial communities clustered into four groups at D0 (**B**), but three groups at D7 and D30, respectively (**C**,**D**). (**E**) Diversity of bacterial communities at the three time points. The Shannon index showed a sharp decrease at D7 compared with D0. (**F**) The top three bacterial genera in the BV populations.

The bacterial composition of these vaginal communities is shown in Fig. 1A. D0 represents the disease condition, so the bacterial compositions were, as previously reported, clustered into four groups, dominated by BVAB1, *Prevotella* spp., *Sneathia* and *Leptorichia,* respectively (Fig. 1B). After treatment for 5 days, the bacterial abundance was obviously reduced, so that the Shannon index, which is a measure of the number of distinct microbes in a community, had decreased significantly. These results suggested that the number of vaginal microbes, as expected, was controlled during the metronidazole treatment (Fig. 1E). In a retrospective study, we separated the patients into two groups according to the ultimate outcome, and investigated the possibility of using the microbial pattern to predict the prognosis. The bacterial microbiota composition pattern was not as expected tightly clustered into two groups associated with the FG and SG groups at D7, but was divided into three groups (Fig. 1C). However, 1 month after the patients had stopped taking the antibiotic (D30), they were separated obviously into two groups, which was consistent with the pre-defined groups based on the Nugent score. This showed the close relationship between the taxonomic categorization based on deep sequencing and the clinical features of BV, which validates the previous report of Sujatha *et al.*^12^. In our analysis, the bacterial composition of the FG group at D30 was similar to that at D0, indicating treatment failure, and the bacterial composition of the other SG group was similar to that of the healthy population (Fig. 1A,D).

We also performed a follow-up survey to examine the microbiota in each individual patient during the treatment. In patients with BV, the diversity of bacteria in all patients was high; however, the structure of the microbiota became simpler and was dominated by *Lactobacillus* when the patients were cured. We also focused on the three most frequent genera in our samples: *Lactobacillus, Prevotella, and Enterococcus* (Fig. 1F). These genera showed significant changes in different treatment periods. *Lactobacillus* was the most commonly observed genus; it can produce various anti-infective agents, and was significantly increased at D7 and in most samples also at D30. However, *Enterococcus* increased at D7, but had decreased at D30. This suggests that *Enterococcus* may be resistant to metronidazole but that it decreases when a new bacterial flora develops, and that it helps to reconstruct a new healthy environment for the vaginal microbiome. *Prevotella*, a potential pathogen involved in BV, was significantly reduced by antibiotic treatment. However, it increased in 27% of patients, and this indicated a poor prognosis.

### Vaginal microbial community used to identify recurrence and new infections

The high recurrence rate of BV after treatment with the currently recommended protocol of metronidazole and clindamycin is a major threat and seriously affects the lives of patients. Based on this observation, we hypothesized that the population with recurrent BV is divided into two groups: one was not cured after the standard treatment although their symptoms resolved, and the other was re-infected. To investigate our hypothesis, we performed analysis of the association between the results at D0 and at D30.

**Figure 2:**
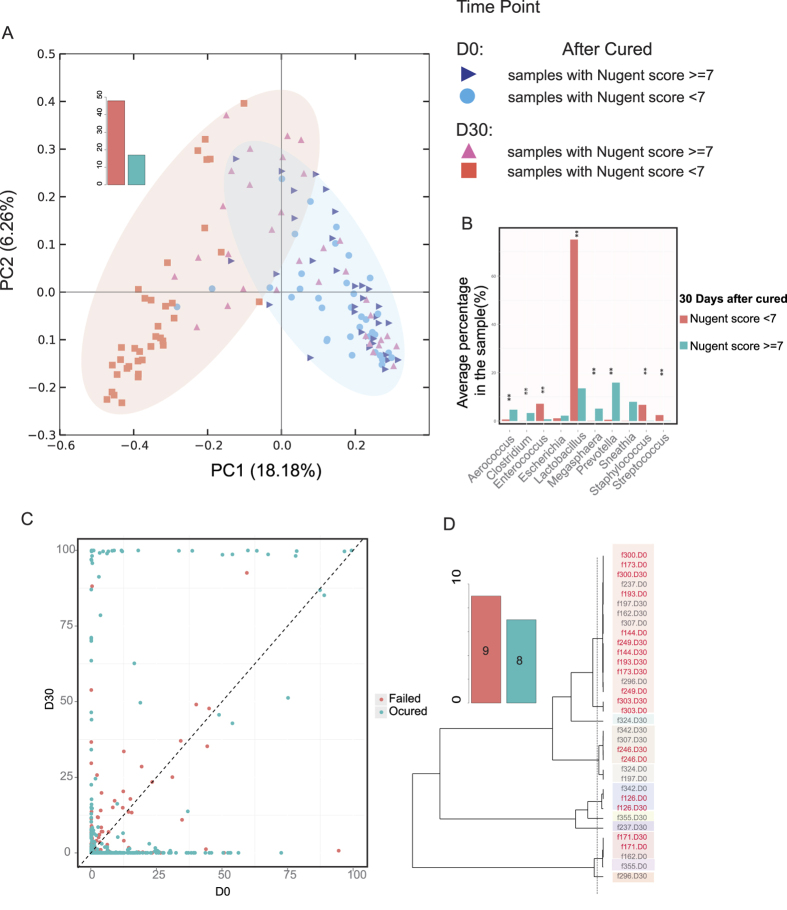
Diversity of the vaginal microbial community and identification of recurrence and re-infection. (**A**) Principal coordinates analysis of the Bray–Curtis dissimilarity among all samples at D0 and D30. Colors were defined according to the Nugent score and the time point of sample collection. The vaginal microbial community showed two patterns, FGP and SGP. (**B**) The top 10 bacterial taxa with different average relative abundances in FGP and SGP at 30 days after treatment. **Indicates *P* *<* *0.05*. (**C**) The collinearity analysis of the relative abundance at D0 and D30. Cyan dots show the bacteria from the SGP and dark pink dots show bacteria from FGP. (**D**) Hierarchical cluster tree of patients in FGP at D30. Nine patients were defined as showing recurrence and eight with re-infection. Red represents the patients with treatment failure caused by recurrence, while gray represents the patients who were re-infected.

Principal coordinates analysis (PCoA) of the Bray–Curtis dissimilarity of all the samples obtained at D30 revealed that the vaginal microbial communities of these patients were divided into two clusters, the FG pattern (FGP) and the SG pattern (SGP) (Fig. 2A). As expected, these robust patterns almost overlapped the previously defined groups FG and SG obtained by the traditional diagnostic criteria, except that two patients at D0 presented a similar bacterial pattern to SGP. This result indicates that these two patients had a microbiota of normal composition, but had clinical symptoms, with a relatively high Nugent score (>7), vaginal fluid pH > 4.5, positive whiff test and the presence of clue cells. The composition of the microbiota is an important factor in causing disease but not the only factor. We compared the composition and relative abundance of bacterial populations in the FGP and SGP groups. Consistent with previous reports, the average relative abundance of *Lactobacillus* was higher in SGP, whereas the composition of *Aerococcus, Clostridium, Sneathia and Prevotella* were significantly lower (Fig. 2B). This result indicates that the pattern of the microbiota can be used to evaluate the outcome of treatment.

Notably, the vaginal bacterial composition of FG patients at D0 and D30 were closely clustered in the PCoA map. This suggested that their microbiota had not changed during treatment, which may indicate recurrence or failure to be cured by metronidazole after 5 days of treatment. To explore this observation further, we mapped the relative abundances of bacteria on D0 to D30, to examine whether they were collinear between these two conditions. When compared with SG, we observed a strong coordinate for the composition of FG, with a correlation coefficient of 0.44 (P = 0.15, Fig. 2C), whereas in SG, the p-value for the association between the two time points was 2e–16. This suggested that at least some patients with BV had no changes in the composition of their bacterial community following treatment with metronidazole.

To determine the outcome of patients with either recurrence of infection surviving after treatment or re-infection, a hierarchical cluster method was used to analyze the similarity of the bacterial class structure (Fig. 2D). Nine cases were clustered in the same clade at D0 and D30, which indicates that they had a similar bacterial composition; this may indicate recurrence of infections that were difficult to treat with metronidazole. In contrast, the bacterial compositions of the other eight patients at these two time points were dispersed in different clades, indicating that they might be re-infections. We further compared their bacterial composition to SG to explore the reason for antibiotic resistance. Unfortunately, limited by the small sample set, we found no significant differences in the relative abundance of bacterial species, especially the dominant species, in these samples.

### Accurate vaginal microbiota map ahead the observation time point of BV to estimate clinical outcome

**Figure 3:**
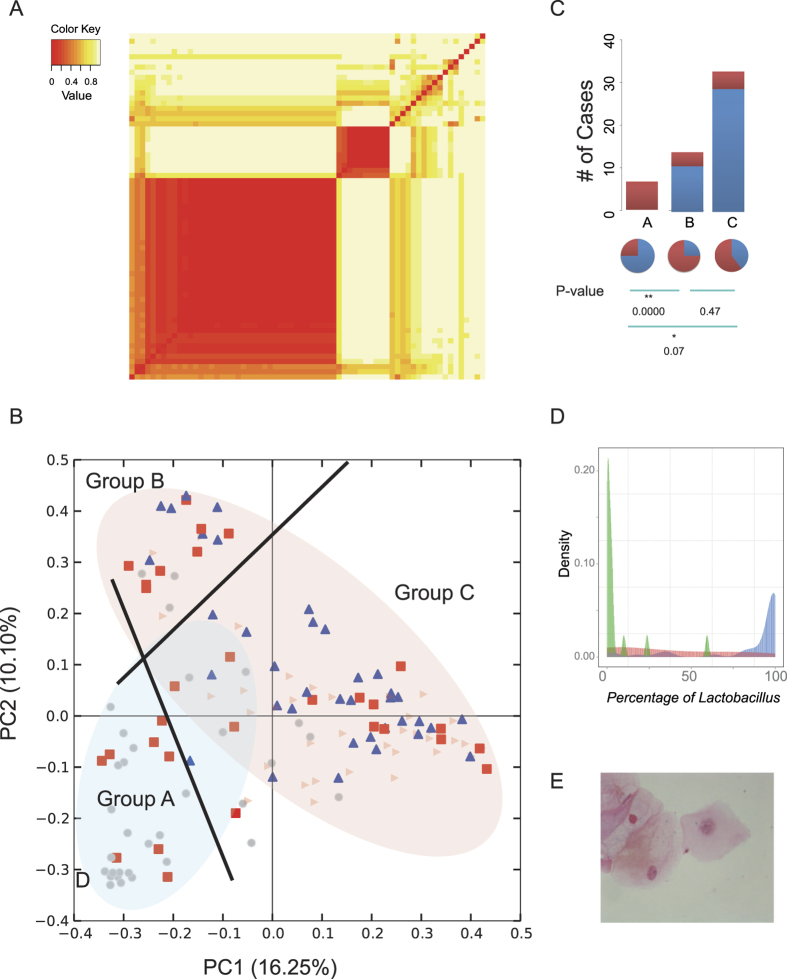
The vaginal microbial community at D7. A heatmap graph (**A**) and principle coordinate analysis (**B**) of the vaginal microbial community at D7. The populations were divided into three separate groups. The red diamond represents patients who showed treatment failure and the blue triangle represents patients with successful treatment. (**C**) The treatment outcomes corresponding to clinical diagnosis at D30 and the recurrence rate in three different groups. The red in the bar plot shows patients with failed treatment outcomes and blue shows successful treatment. Blue in the pie chart represents the rate of recurrence. *Indicates *P* < *0.1* and **indicates *P* < *0.01.* (**D**) The relative abundance of *Lactobacillus* at D7. (**E**) Low density of microbe in vaginal, a microscope view with Gram-stained.

The Nugent score and Amsel’s diagnostic criteria describe the patient condition well when the patient does not use antibiotics, but they are less useful if all bacteria are resistant or in patients with negative results. Using deep sequencing, we used an extra detection point to observe the therapeutic effects and prognosis of the disease. Day 7 was selected as the detection point to observe the clinical efficiency of treatment by antibiotics, and the microbial composition at the point was scanned. We first compared these three groups with we previously defined groups by metabiota data in D30, SGP and FGP. From the heatmap of the bacterial composition of these samples, we could clearly categorize the patients into three groups, which were defined as groups A, B, and C (Fig. 3A). These three groups were dramatically projected in the principle coordinates map (Fig. 3B). Group A all belonged to FGP, whereas groups B and C both belonged to SGP, but were divided into two distinct parts (Fig. 3B). Group C was tightly clustered. These results suggest that the composition of the microbiota at D7 has the ability to predict the final outcome of the disease. More importantly, these vaginal microbiota compositions detected using the deep sequencing method could be identified in low concentrations of bacteria after antibiotic treatment, and were still able to be used to predict the prognosis.

We then examined the relationship between the clinical outcomes defined by the Nugent score and the different groups that were automatically defined by the bacterial taxa at these time points. The Nugent scores were highly consistent with the three groups (Fig. 3C). All patients in group A finally failed to be cured. Patients in group B have higher probability to recur in groups. This suggested that, coupled with NGS based genus-level classification, early detection at D7 can predict the outcome of BV. We also selected the dominant genus in the normal flora, *Lactobacillus*, as a control, of which the relative abundance was low, as expected (Fig. 3D). In our analysis, the patients in group A all scored more than 7 at D30, which suggested that these patients may not have been cured, although their symptoms were resolved and they had a Nugent score of less than 7 at the initial stage.

Furthermore, we used these microbiome-based patterns to predict the recurrence of disease, and the results were the same as the final result at D30. Additionally, we noticed that the three groups classified on the basis of the microbiome represented different recurrence rates and cure rates of clinical treatment. In group A, all patients failed to respond to treatment with metronidazole, and 75% patients underwent recurrence. In group B and group C, most of the patients were cured, with a relatively low recurrence rate (Fig. 3C).

## Discussion

The human vaginal microbiota is highly variable among women, as shown by culture independent methods, and although all studies agree that the genus *Lactobacillus* is the predominant member of the vaginal microbiota, communities can range from 100% *Lactobacillus* to no *Lactobacillus* species present^20^. However, the bacterial composition in BV is strongly associated with the clinical features, with a certain tightly clustered pattern, which is separated into a significant cluster when compared with patients without BV, and is dominated by *Aerococcus*, *Clostridium*, *Sneathia* and *Prevotella.* In previous studies, the analysis of the vaginal flora after treatment was used to indicate the prognosis of treatment, and it presented a strong association with the clinical features. In this study, we used the differences in bacterial composition before and after treatment to reflect the possible role of re-infection and relapse of BV, which has aroused wide concern. In terms of sequencing, the vaginal microbiome of five patients diagnosed with BV again after one month had significantly changed. The degree of similarity between D0 and D30 was 0.63%, 0.22%, 0.75%, 1.57%, and 0.43%, respectively, in these five patients. We considered that this indicated re-infection. On the contrary, 10 samples showed a degree of similarity between D0 and D30 of more than 80%, ranging from 83.62–98.82%, which indicated BV relapse. This result suggests that deep sequencing based microbiome analysis can succeed in identifying modification of the vaginal microbiome, which helps to identify cases of recurrence or re-infections occurring during treatment. However, limited by the case number, we do not carry out a more complete epidemic surveillance to adjust the sufficient time point of this detection.

Besides that, the analysis of the vaginal flora can also predict the recurrence of BV by the comparison of the changes of microbiota. Bacterial vaginosis promotes the transmission of sexually transmitted diseases, including gonorrhea, chlamydia, syphilis, trichomoniasis, human immunodeficiency virus (HIV) and human papillomavirus (HPV)^21^. Women with BV have a complex vaginal bacterial community with increased species richness, which is decreased by treatment; however, they are vulnerable to the recurrence of BV when they have abnormal vaginal communities. Recruitment of more women and increasing the number of samples will extend our understanding of the high recurrence rate of BV. Our studies will be focused on patients who were not cured, although their symptoms resolved, but were not infected again during treatment. For example, we may identify which bacteria did not change during treatment, and these may be resistant to the antibiotic used. Physicians will therefore be able to adjust the therapeutic regimen by adding specific probiotics or prolonging the treatment course individually to reduce the recurrence rate.

Nugent scores are based on weighted counts of different cellular morphotypes: *Lactobacilli*, *Gardnerella vaginalis* or *Bacteroides* (small Gram-variable rods or Gram-negative rods), and curved Gram-variable rods. The resulting scores range from 0–10, with those of 7 and higher considered to be indicative of BV, whereas scores of 4–6 and 3 or less are considered intermediate and normal, respectively. This criterion is widely used in BV diagnosis. In this study, we used this method as one of comparable factors. *Lactobacillus* dominated in group C after metronidazole treatment, accounting for 87%; 34 patients were cured eventually, with four relapsing. *Lactobacillus* plays an important role in the outcome of BV treatment. In most cases, a large amount of *Lactobacillus* can reduce the recurrence rate. The healthy human vagina is physiologically dominated by *Lactobacillus* species, which are responsible for protecting the host from bacterial infections. However, the four cases that were dominated by *Lactobacillus* showed relapse at one month. Previous studies have shown that human habits and practices, including personal hygiene, methods of birth control and sexual behaviors, also exert a strong influence on the progress of recovery^1^. Also, the Nugent score leads to a bias when bacterial abundance is low. We observed three situations using light microscopy at the end of treatment: the normal flora, a few bacteria (or no bacteria) (Fig. 3E), or a large decrease in diversity and density of the flora and a large number of cocci. These observations were associated with the results obtained from pyrosequencing. The slides with a few bacteria could be explained in two ways. The first is that the there are insufficient vaginal bacteria in some smears, owing to human factors. These biases are not easy to overcome in situations with a low abundance of bacteria. The other reason is that the antibiotics have an impact on all bacterial species in the vagina, including *Lactobacillus* and other anaerobic bacteria. In this study, there are no means to predict the prognosis of BV from slides containing few bacteria because metronidazole has eliminated all susceptible bacteria. The Nugent score is unable to reflect the bacterial species accurately in this situation, which is why few clinicians advise patients to return immediately after using an antibiotic. However, the sequence-based techniques can better represent the vaginal bacterial composition and structure, which gives the physicians more information for earlier evaluation and treatment of the BV. It can therefore reduce the risk of recurrence of BV, and reduce the complications resulting from BV in women of reproductive age.

The vaginal microbiome of Cluster A did not change obviously, and did not revert to normal. The result obtained from slides showed that the Nugent scores of all samples at 6–8 days were below 7. Then metronidazole treatment was ceased. In fact, at the molecular level, metronidazole exerts no influence on the major bacteria that cause BV, including *Gardnerella*, *Provetella*, *Sneathia and Atopobium*. We postulate that these species are resistant to metronidazole or that the course of treatment is insufficient.

In conclusion, although patients with BV have a complete Nugent score and Amsel’s diagnostic criteria to categorize the disease condition by light microscopy, there are still some problems in precisely describing the composition of the bacterial community. Deep sequencing analysis provides a clearer picture of the bacteria community, which extends our understanding of the BV process and allows detection of recurrence and treatment failure, which may indicate bacterial resistance to metronidazole. Furthermore, based on this method, we may be able to detect BV earlier, so that patients may receive more suitable treatments.

## Materials and Methods

### BV cases

We recruited 192 women actively treated for BV at Peking University First Hospital from September 2012–July 2013. The women were not pregnant, were of reproductive age, ranging from 18–53 years, and had regular menstruation. Women with any of the following exclusion criteria were excluded from a test-of-cure visit for BV: less than 18 years of age, pregnancy, menstruation, sexual intercourse within 24 h, the use of antibiotics in the last month and any intravaginal product in the last 24 h, the presence of yeast on Gram stain or *Trichomonas vaginalis* infection. Patients volunteered to participate in the study and gave written informed consent. The study was approved by the ethics committee of Peking University First Hospital College, Beijing, China, and was strictly carried out in accordance with the approved guidelines. Women with BV were treated with a single 5-day regimen of intravaginal metronidazole gel (37.5 mg daily) and were asked to return at 6–8 days and 1 month for a test-of-cure examination.

### Sample collection

Vaginal samples (one for Gram staining and one for bacterial genomic DNA extraction) were collected at the initial visit, at 6–8 days, and at 1 month after 5 days of treatment with intravaginal metronidazole gel. At each visit, two vaginal swabs were placed in the vagina at a standard anatomical site (lateral vaginal wall). The first swabs were stored in 1 mL of PBS and frozen upright on dry ice until transported to the laboratory, where they were stored at −80 °C. The first vaginal swab of vaginal fluid was used for genomic DNA extraction. The second swab was rolled onto a microscope glass slide that was Gram-stained, and then scored using Nugent criteria by two experienced microscopists.

BV was diagnosed using Amsel’s criteria and confirmed on a Gram-stained vaginal smear using the scoring method described by Nugent^22^,^23^. According to the modified Amsel criteria, BV was diagnosed when three of the following were present: a thin homogeneous discharge, elevated vaginal pH above 4.5, release of amines on the addition of 10% potassium hydroxide to vaginal fluid, and the presence of “clue” cells. The resulting Nugent scores ranging from 7–10 were considered to be indicative of BV. The patients with BV who achieved clinical cures were evaluated according to Amsel criteria in combination with Nugent scores (<4).

### DNA extraction, sequencing and data analysis

Vaginal specimens were collected, stored and processed for bacterial genomic DNA extraction using the method described by Ling *et al.*^24^. Genomic DNA was extracted using the QIAamp DNA Mini Kit (QIAGEN, Hilden, Germany). In brief, 20 μl proteinase K solution (20 mg/ml) and 100 mg zirconium beads (0.1 mm) were added to the pellet. The mixture was agitated three times on a Mini-Beadbeater (BioSpec Products Inc., Bartlesville, Oklahoma, USA); buffer AL was added to the mixture, which was then incubated for 10 min at 70 °C. Next, 200 μl ethanol (96%) was added. This mixture was loaded onto the QIAamp Mini spin column and centrifuged at 8,000 *g* for 1 min. The column material was washed with the first washing buffer (buffer AW1, 500 μl) and with the second washing buffer (buffer AW2, 500 μl) provided with the kit. Finally, DNA was eluted with 100 μl of buffer AE. The integrity and size of the extracted DNA were checked by electrophoresis on 1% agarose gels containing 0.5 mg/ml ethidium bromide. The DNA concentration was determined using a NanoDrop ND-2000 spectrophotometer.

The bacterial species composition and abundance in the vaginal communities were determined using culture-independent methods. The V1–V3 hypervariable regions of the 16 S rRNA gene (16S rDNA) were amplified using an optimized primer set comprising 27F and 534R^25^. Amplicon pyrosequencing was performed using standard 454/RocheGS-FLX protocols^26^. All sequences were trimmed according to the quality. To pass, a sequence had to meet the following criteria: 1) at least 200 bp in length and no more than two undetermined bases; 2) an average of q25 over a sliding window of 25 bp. If the read quality dropped below q25, it was trimmed at the first base pair of the window then reassessed for the length criterion; 3) a perfect match to a barcode sequence and the 16 S rDNA primer; and 4) presence of the 534R 16S primer sequence used for amplification. Sequences were binned on the basis of sample-specific barcode sequences and trimmed by removal of the barcode and primer sequences. On average, 5% of the sequence reads did not pass this quality-control procedure. The raw data has been deposited in the GenBank database (Accession number: SRP066130).

Each processed 16S rDNA sequence was classified using the RDP Naïve Bayesian Classifier^27^. All reads were classified to the genus level using the QIIME software package^28^. The candidate sequences representing each taxon were downloaded from the RDP database. The composition of each genus was estimated according the specific reads presented in the sample. The genera which were contained in at least two samples and at least at 0.1% were considered to be present in these samples. PCoA analysis of different samples was calculated according to the phylogenetic Bray–Curtis metric with weighted uniFrac^29^. The correlation between the compositions at D0 and D30 was calculated using Spearman’s rho statistic, which was used to estimate a rank-based measure of association. Raup–Crick dissimilarity was used to evaluate the genus sampling probability, to estimate its recurrence (vegan, R package).

## Additional Information

**How to cite this article**: Xiao, B. *et al.* Predictive value of the composition of the vaginal microbiota in bacterial vaginosis, a dynamic study to identify recurrence-related flora. *Sci. Rep.* **6**, 26674; doi: 10.1038/srep26674 (2016).

## Supplementary Material

Supplementary table 1
