# Supplementary material for: Predictive value of the composition of the vaginal microbiota in bacterial vaginosis, a dynamic study to identify recurrence-related flora
Source: Sci Rep. 2016 Jun 2;6:26674. doi: 10.1038/srep26674 (PMC4890590; doi:10.1038/srep26674)
Supplement: Supplementary table 1 [file srep26674-s1.pdf]

Predictive value of the composition of the vaginal microbiota in bacterial vaginosis, a dynamic study to identify recurrence-related flora

Bingbing Xiao, Xiaoxi Niu, Na Han, Ben Wang, Pengcheng Du, Risu Na, Chen Chen, and Qinping Liao

Supplementary table 1: The numbers of OTUs in individual samples from 68 patients at three different time points: Day 0 (D0, before treatment), Day 7 (D7, stop using the antibiotics) and Day 30 (D30, the 30-day follow-up visit).

Patient ID	# of OTUs			Outcome
	Day 0	Day 7	Day 30	
118	289	No data	346	Failed
126	677	264	275	Failed
144	372	276	349	Failed
162	392	92	307	Failed
171	182	148	168	Failed
173	520	85	571	Failed
193	366	120	274	Failed
197	483	54	380	Failed
237	499	112	498	Failed
246	195	117	165	Failed
249	339	249	309	Failed
296	276	90	137	Failed
300	617	185	465	Failed
303	272	58	148	Failed
307	345	83	364	Failed
309	272	No data	121	Failed
324	200	387	114	Failed
342	469	70	393	Failed
355	137	132	432	Failed
30	433	144	100	Cured
70	483	66	68	Cured

111	304	210	87	Cured
124	411	76	60	Cured
127	289	74	130	Cured
130	706	77	111	Cured
151	426	91	86	Cured
156	247	75	157	Cured
166	420	98	88	Cured
167	393	No data	54	Cured
168	275	105	346	Cured
170	273	109	272	Cured
175	352	78	84	Cured
182	209	134	72	Cured
188	419	59	71	Cured
191	427	78	93	Cured
196	380	74	118	Cured
198	581	82	123	Cured
205	440	189	84	Cured
209	561	71	99	Cured
230	631	95	78	Cured
232	365	92	231	Cured
238	325	118	153	Cured
243	208	137	132	Cured
244	227	105	91	Cured
247	432	132	377	Cured
248	104	79	104	Cured
250	262	196	73	Cured
265	319	72	56	Cured
267	437	109	69	Cured
270	528	68	158	Cured
272	364	207	127	Cured
273	304	71	75	Cured
275	406	81	66	Cured
278	266	104	238	Cured
288	400	96	75	Cured
292	270	66	182	Cured
293	151	162	102	Cured
294	324	136	179	Cured
301	208	56	130	Cured
311	259	129	285	Cured
313	309	177	118	Cured
318	298	82	202	Cured
322	339	79	67	Cured
328	603	90	209	Cured
332	460	120	131	Cured

	339	238	128	221	Cured
	340	427	73	123	Cured
	356	321	99	163	Cured
